# Exploring the Aβ_1-42_ fibrillogenesis timeline by atomic force microscopy and surface enhanced Raman spectroscopy

**DOI:** 10.3389/fmolb.2024.1376411

**Published:** 2024-06-14

**Authors:** Panagis Polykretis, Cristiano D’Andrea, Martina Banchelli, Liliana Napolitano, Roberta Cascella, Marella de Angelis, Paolo Matteini

**Affiliations:** ^1^ Institute of Applied Physics “Nello Carrara”, National Research Council, Sesto Fiorentino, Italy; ^2^ Department of Experimental and Clinical Biomedical Sciences, Section of Biochemistry, University of Florence, Florence, Italy

**Keywords:** amyloid-β peptide, fibrillogenesis, Alzheimer’s disease, neurodegeneration, AFM, SERS

## Abstract

**Introduction:** Alzheimer’s disease (AD) is a progressive debilitating neurological disorder representing the most common neurodegenerative disease worldwide. Although the exact pathogenic mechanisms of AD remain unresolved, the presence of extracellular amyloid-β peptide 1-42 (Aβ_1-42_) plaques in the parenchymal and cortical brain is considered one of the hallmarks of the disease.

**Methods:** In this work, we investigated the Aβ_1-42_ fibrillogenesis timeline up to 48 h of incubation, providing morphological and chemo-structural characterization of the main assemblies formed during the aggregation process of Aβ_1-42_, by atomic force microscopy (AFM) and surface enhanced Raman spectroscopy (SERS), respectively.

**Results:** AFM topography evidenced the presence of characteristic protofibrils at early-stages of aggregation, which form peculiar macromolecular networks over time. SERS allowed to track the progressive variation in the secondary structure of the aggregation species involved in the fibrillogenesis and to determine when the β-sheet starts to prevail over the random coil conformation in the aggregation process.

**Discussion:** Our research highlights the significance of investigating the early phases of fibrillogenesis to better understand the molecular pathophysiology of AD and identify potential therapeutic targets that may prevent or slow down the aggregation process.

## 1 Introduction

The American Alzheimer’s Association has estimated that ca. 55 million people worldwide are living with Alzheimer’s disease (AD) and other neurodegenerative disease leading to dementia (Alzheimer’s Association, 2023). AD is a devastating degenerative brain disease and the most common form of dementia. The pathogenic mechanism of AD is not fully understood; however, the aggregation of two different proteins in the parenchymal brain constitute the main hallmarks of the disease: the amyloid-β peptide, which forms extracellular amyloid plaques, and the tubulin-associated unit (tau) isoforms, which form intracellular hyperphosphorylated neurofibrillary tangles (Vermunt et al., 2019; Bistaffa et al., 2020; Pons and Rivest, 2022). AD is typically diagnosed *in vivo* when irreparable brain damage has occurred, while the definite diagnosis of AD can only be made post-mortem, upon the detection of the aforementioned aggregates through brain autopsy (Bistaffa et al., 2020). Therefore, one of the primary goals of the research in this field is to develop a sensitive, reproducible and cost-effective approach for the identification of diagnostic biomarkers, particularly in the early phases of AD, when a diagnosis based on cognitive symptoms is more uncertain and therapeutic intervention could be more efficacious (Jack et al., 2018; Bellomo et al., 2021; Bellomo et al., 2024).

The presence of amyloid-β in human brains and cerebrospinal fluid (CSF) throughout life is physiological (Broersen et al., 2010), and although its exact function is still unknown, the amyloid-β peptide is produced when β- and γ-secretase sequentially cleave the amyloid precursor protein (APP) (Müller et al., 2017; Steiner et al., 2018). APP has been found to play a role in the brain’s neuronal growth promoting the migration of neurons during the early stages of brain development (O’Brien and Wong, 2011). Furthermore, APP is involved in a variety of biological processes, including synapse maintenance, plasticity, transcriptional regulation and neuroprotection (Zheng and Koo, 2011). β-secretase initially cleaves APP in its extracellular domain, producing the C-terminal APP fragment (C99), which is then cleaved by γ-secretase at different sites releasing in the extracellular space amyloid-β species ranging from 37 to 43 amino acids. Longer amyloid-β variants, such as Aβ_1-42_, exhibit a high propensity for aggregation and eventually undergo to fibrillation process (Steiner et al., 2018). The non-amyloidogenic cleavage of APP by α-secretase prevents the formation of the abovementioned species and thus the fibrillation process. Mutations in the human APP gene, in proximity to the γ-secretase cleavage site, could be responsible for the formation of the amyloid-β species with higher propensity to aggregate (Chen et al., 2017). Furthermore, age-dependent loss of regulatory mechanisms, caused by long-term inflammatory conditions, results in the dysregulation of cellular systems involved in the clearance and degradation of misfolded or damaged neuronal proteins (Höhn et al., 2020). The failure of proteostasis thus promotes the accumulation of aberrant protein aggregates that may lead to the onset of AD (Krstic and Knuesel, 2013; Bigi et al., 2024a). In the last decades, small oligomers of Aβ_1-42_, formed early during the aggregation process or released from mature fibrils, have acquired increasing importance as primary toxic species in AD pathogenesis (Kayed et al., 2004; Benilova et al., 2012; Bigi et al., 2022; Limbocker et al., 2023). Moreover, amyloid-β aggregates were identified in the CSF (Kasai et al., 2013; Savage et al., 2014; De et al., 2019; Sun et al., 2021; Nirmalraj et al., 2023; Bigi et al., 2024b).

Atomic force microscopy (AFM), due to its capability for the nano-structural analysis of individual macromolecules, has extensively demonstrated to be a powerful technique for the study of the amyloid-β aggregation as well as for the morphological characterization of the amyloidogenic aggregates, such as oligomers, protofibrils and fibrils (Harper et al., 1997a; Harper et al., 1997b; Blackley et al., 1999; 2000; Kowalewski and Holtzman, 1999; Kowalewski and Holtzman, 1999; Dahlgren et al., 2002; Arimon et al., 2005; Bartolini et al., 2011; Moores et al., 2011; Jiang et al., 2012; Drolle et al., 2014; Breydo et al., 2016; Stylianou et al., 2019; Nirmalraj et al., 2020). The combination of AFM with circular dichroism spectroscopy (CD) and the measurement of the ζ-potential allowed to identify two different aggregation pathways, the amorphous and the fibrous, monitoring variations in the secondary structures and correlating each pathway with the colloidal stability of the aggregation intermediates, which is essential to the fibrillation process (Jiang et al., 2012). Moreover, the aforementioned study monitored the changes in the surface charges of the amyloid-β molecules due to Cu^2+^ binding, demonstrating that the metal ion enhances the amorphous aggregate formation. The binding of Cu^2+^ to Aβ_1-42_ has been also exploited as a probe for estimating the intramolecular distances in the oligomers by double electron-electron resonance (DEER), in combination with AFM and surface enhanced Raman spectroscopy (SERS) (Banchelli et al., 2021). SERS is a powerful optical technique which allows to obtain the chemo-structural characterization of molecular species, by providing a label-free detection at sub-micromolar concentrations together with a spectral fingerprint information (D’Andrea et al., 2018; D’Andrea et al., 2023; Banchelli et al., 2019; Banchelli et al., 2020; Polykretis et al., 2022).

In this work we have examined the Aβ_1-42_ fibrillogenesis timeline using AFM, Thioflavin T (ThT) fluorescence assay and SERS in order to characterize the aggregation intermediates from a morphological and spectroscopic point of view. In particular, we have monitored the progressive variations in their secondary structure during all phases of fibrillation, from the first minutes until the formation of mature fibrils. This approach allowed to identify protofibrillar species at early-stages of aggregation, which form peculiar macromolecular networks over time.

## 2 Materials and methods

### 2.1 Preparation of amyloid-β fibrils

Aβ_1-42_ fibrils have been prepared as previously reported (Ladiwala et al., 2012). Briefly, the lyophilised peptide (Bachem, Bubendorf, Switzerland) was dissolved in 100% hexafluoro-2-isopropanol (HFIP) to 1 mM, and the solvent was then evaporated under gentle nitrogen steam. To obtain Aβ_1-42_ fibrils, the peptide was resuspended in 50 mM NaOH at 1 mg/mL, diluted in PBS (PAN-Biotech, Aidenbach, Germany) at 50 µM (pH of the final solution = 12.1), and the sample was incubated at 25 °C in a PCR thermal cycler (BioRad T100). Aliquots were taken at specific incubation times: Immediately after the initiation of Aβ_1-42_ fibrillation, after 60 min, 120 min, 240 min, 480 min, 24 h and 48 h, indicated as 0’, 60’, 120’, 240’, 480’, 24 h and 48 h, respectively.

### 2.2 AFM

Hydrophilic mica has been selected as substrate for the AFM experiments as it can be easily cleaved to produce clean, atomically smooth surfaces with a roughness of ∼0.2 nm. After the mica was newly cleaved, 3 μL of the aliquot taken from each incubation step was deposited on top and dried at 37 °C for 90 min. The samples were rinsed two times with MilliQ water (100 μL) in order to remove salts and debris and then dried at 37°C. Each sample was imaged using a JPK NanoWizard III Sense (Bruker, Berlin, Germany) scanning probe microscope operated in tapping mode. Single-beam uncoated silicon cantilevers (HQ:NSC15/Cr-Au BS, MikroMash) with a force constant of 40 N/m, and a tip radius of <8 nm, working at a resonant frequency range between 230 and 300 kHz, were employed. The scan rate used during the measurements ranged from 0.4 to 1 Hz and the number of pixels was set to 1024 × 1024. The JPK Data Processing software was used for the data analysis and the creation of the topographic images, while the measured widths of the fibrils were corrected for the tip-induced broadening as previously reported (D’Andrea et al., 2018).

### 2.3 ThT fluorescence assay

Monomeric Aβ_1-42_ was incubated in 50 mM NaOH at 1 mg/mL, diluted in PBS at 50 µM as previously reported. Samples were prepared with a final concentration of 25 μM ThT dye, gently vortexed, and pipetted into nonbinding surface black 96-well plates (Greiner Bio-One, Frickenhausen, Austria) in quintuplicates. The plates were read in a BioTek SynergyTM H1 Hybrid Multi-mode reader (Agilent, Santa Clara, United States) at 25°C. The excitation and emission wavelengths were set to 440 and 485 nm, respectively. Buffer-only values were not subtracted from the sample readings but shown in the final graph. Readings were taken every 2 min. Data were plotted using GraphPad Prism version 5.00 for Windows (GraphPad Software, San Diego, CA, United States). As a control, we also performed a ThT experiment on solutions containing only 50 mM NaOH and PBS with no Aβ_1-42_ monomer.

### 2.4 SERS

All incubation products were analyzed using a SERS substrate based on networks of silver nanowires (AgNWs), as previously reported (Banchelli et al., 2019; Barucci et al., 2021; D’Andrea et al., 2023). A volume of 2 µL of each incubation product was deposited on a SERS-active spot, dried at RT, rinsed twice with 2.5 µL of MilliQ water for 1 min in order to remove any residual trace of PBS buffer, and finally dried at RT for 90 min. The SERS spectra were acquired using a LabRAM HR Evolution spectrometer (Horiba, Lille, France) working in back-scattering geometry equipped with a Synapse Plus CCD detector (Horiba, Lille, France), an excitation laser source with wavelength of 633 nm, focused through a ×50 objective (Olympus, Hamburg, Germany) and laser power of 10 µW. For each sample a total of 50 spectra on different positions within an area of 600 × 400 μm^2^ were acquired illuminating the sample for 1 s of integration time. The spectrometer was calibrated in wavelength using the first-order Raman peak (520.8 cm^-1^) in the spectrum recorded from a bulk crystalline silicon sample. To mitigate eventual signal fluctuations in the signals resulting from operational factors such as local inhomogeneities of the AgNWs substrate, variations in laser focusing or background autofluorescence, and to appreciate small signal changes, the data were pre-processed using LabSpec 6 software (Horiba, Lille, France). In particular, adhering to an established analytical protocol (Krafft et al., 2017; Barucci et al., 2021), averaged spectra derived from 50 acquisition on each sample were corrected for cosmic ray spikes, smoothed, baseline-corrected via polynomial fit, and normalized to the spectral area. Subsequently, a multi-peak fitting procedure employing Gauss-Lorentz functions was performed to accurately fit the Raman amide I band (1590-1720 cm^-1^) of Aβ_1-42_, enabling the extraction of details pertaining the secondary structure of amyloid aggregates.

## 3 Results

### 3.1 AFM

**FIGURE 1:**
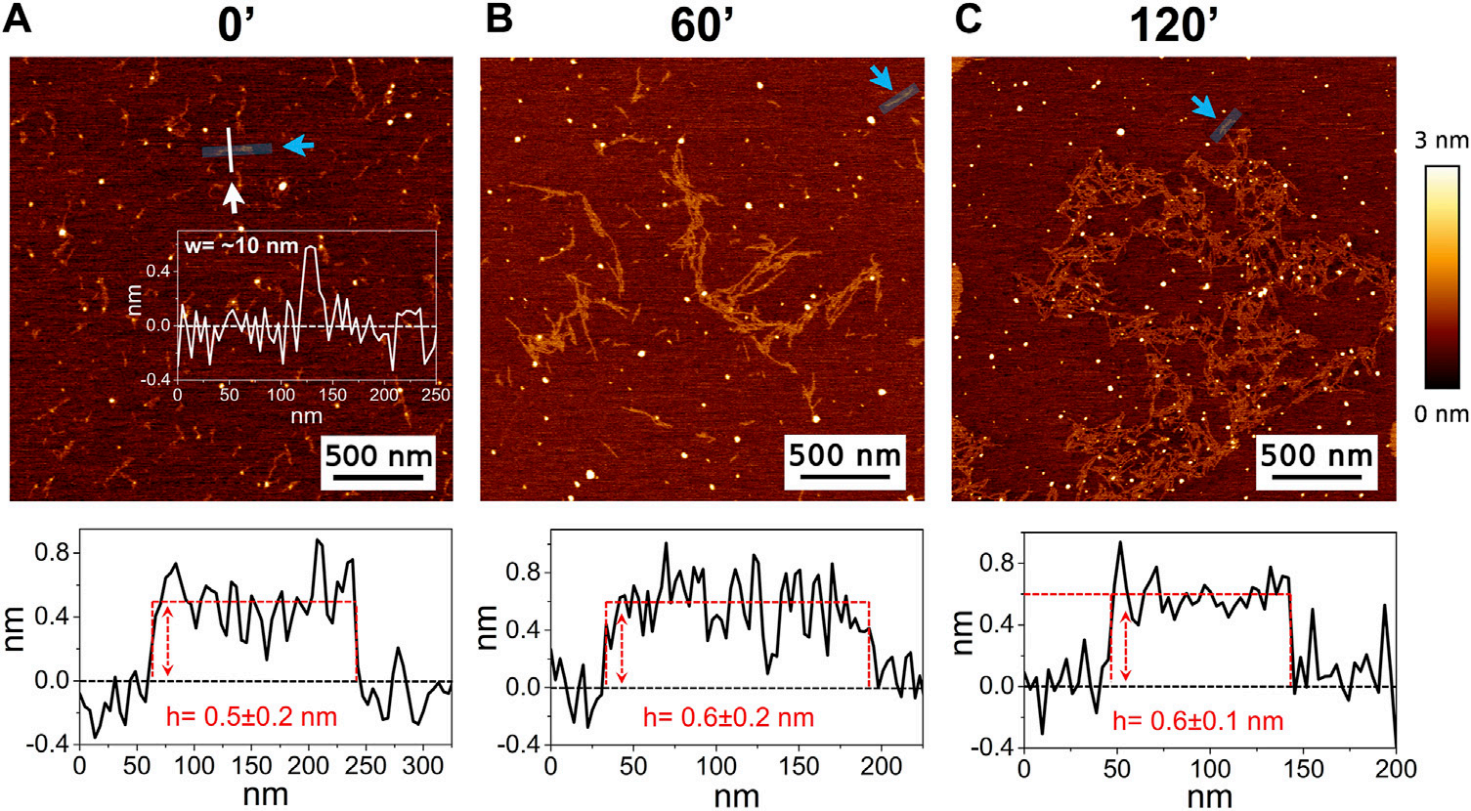
Representative AFM height images acquired on Aβ_1-42_ that was incubated for **(A)** 0 min (immediately after the initiation of Aβ_1-42_ fibrillation), **(B)** 60 min and **(C)** 120 min (the colour-coded height bar is shown beside). The height profiles and the mean height values obtained by measuring along the cyan lines (indicated by the cyan arrows) are displayed beneath the corresponding image (where the ordinate axis indicates the height and the abscissa axis indicates the length). **(A)** also displays the representative width of a protofibril as measured along the white line.

**FIGURE 2:**
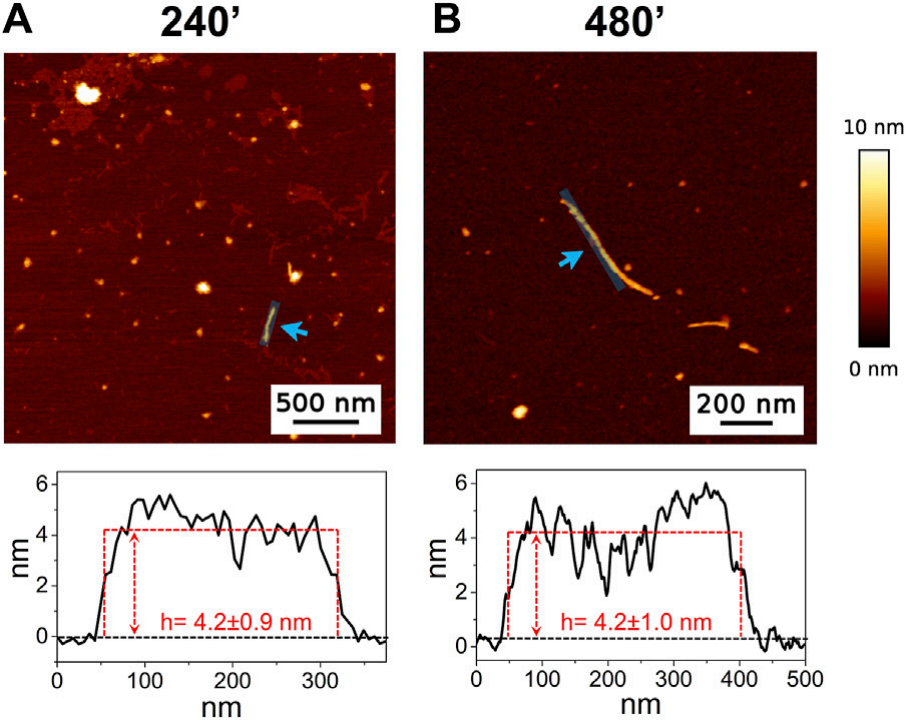
Representative AFM height images acquired on Aβ_1-42_ that was incubated for **(A)** 240 min and **(B)** 480 min (the colour-coded height bar is shown beside). The height profiles and the mean height values obtained by measuring along the cyan lines (indicated by the cyan arrows) are displayed beneath the corresponding image (where the ordinate axis indicates the height and the abscissa axis indicates the length).

**FIGURE 3:**
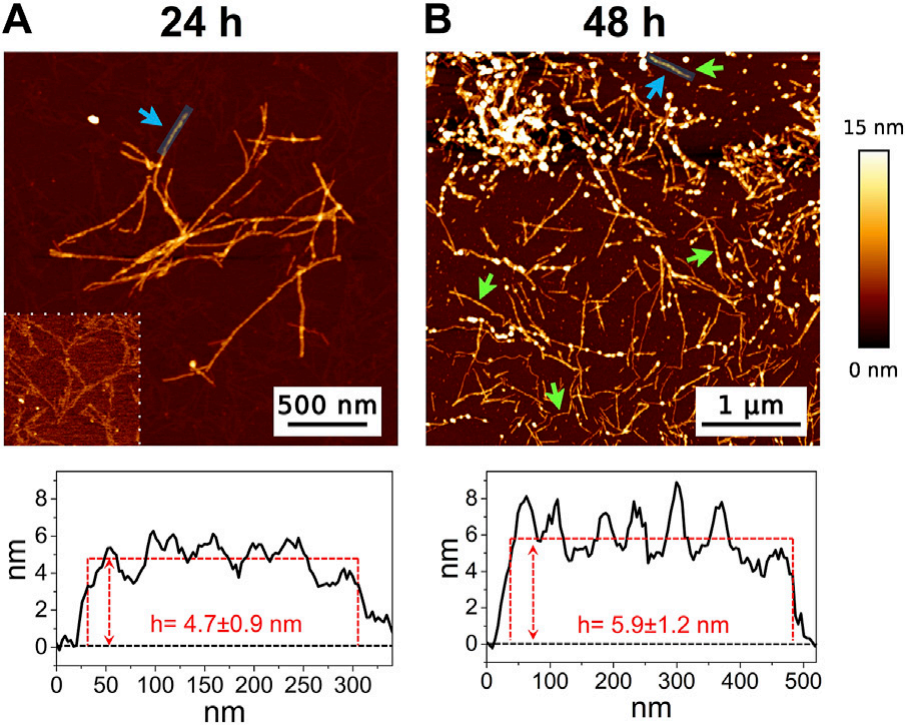
Representative AFM height images acquired on Aβ_1-42_ that was incubated for **(A)** 24 h and **(B)** 48 h (the colour-coded height bar is shown beside). The height profiles and the mean height values obtained by measuring along the cyan lines (indicated by the cyan arrows) are displayed beneath the corresponding image (where the ordinate axis indicates the height and the abscissa axis indicates the length). The panel on the bottom left of figure **(A)** has a narrower height range (0–2.5 nm) to highlight the presence of the protofibrils in the background. The green arrows in figure **(B)** indicate some mature fibrils displaying the periodic helical twist.

The high-resolution AFM imaging on Aβ_1-42_ was collected over time, allowing the morphologic characterization of the fibrillogenesis process throughout its whole timeline and the identification of key structural features of the aggregation species. The presence of small globular aggregates, compatible with oligomeric Aβ_1-42_ assemblies, is observed since the initial incubation times (Figure 1), in accordance with previous studies (Blackley et al., 1999; 2000; Banchelli et al., 2020). Furthermore, short protofibrils with an average length of ∼85 nm, ∼8 nm of width and ∼0.5 nm of height were identified immediately after the initiation of aggregation (Figure 1A). The protofibrils’ extremely short height approaches the mica’s intrinsic roughness (∼0.2 nm), and this may have an impact on the height measurement error. After 60 min of incubation, these protofibrils did not significantly grow in size, but they appeared to be densely connected by probably random interactions (Figure 1B). The aforementioned protofibrils formed a macromolecular network whose area gradually increased at longer incubation times (above 120 min) (Figure 1C). After 240 min of incubation, larger size fibrils with a height of ∼4.2 nm were observed (Figure 2A), which gradually increased in length over time (Figure 2B). After 24 h of incubation, the sample exhibited mature amyloid fibrils with lengths ranging from hundreds of nm to 1.5–2 μm and heights of ∼4.7 nm (Figure 3A). Remarkably, the mature amyloid-β fibrils started displaying the periodical twist that characterizes the formation of helical fibrils. This structural feature has been previously documented on Aβ_1-42_ (Drolle et al., 2014), Aβ_1-40_ (Harper et al., 1997a) and other proteins that form amyloid fibrils (Khurana et al., 2003; Adamcik and Mezzenga, 2012; Lutter et al., 2022). Notably, the mature fibrils were frequently observed lying above the previously described macromolecular network of protofibrils (Figure 3A panel on the bottom left). This indicates that, following a 24 h incubation, the two types of aggregated species were still coexisting, and their spatial proximity suggests that the mature fibrils represent a later developmental stage. Finally, after 48 h of incubation, mature fibrils became the predominant species within the sample, and were characterized by a slight increase in height and a random interaction with large globular aggregates, likely deriving from the amorphous aggregation pathway of Aβ_1-42_ (Figure 3B). Some mature fibrils displaying the periodical helical twist are indicated by green arrows in Figure 3B.

### 3.2 ThT fluorescence assay

**FIGURE 4:**
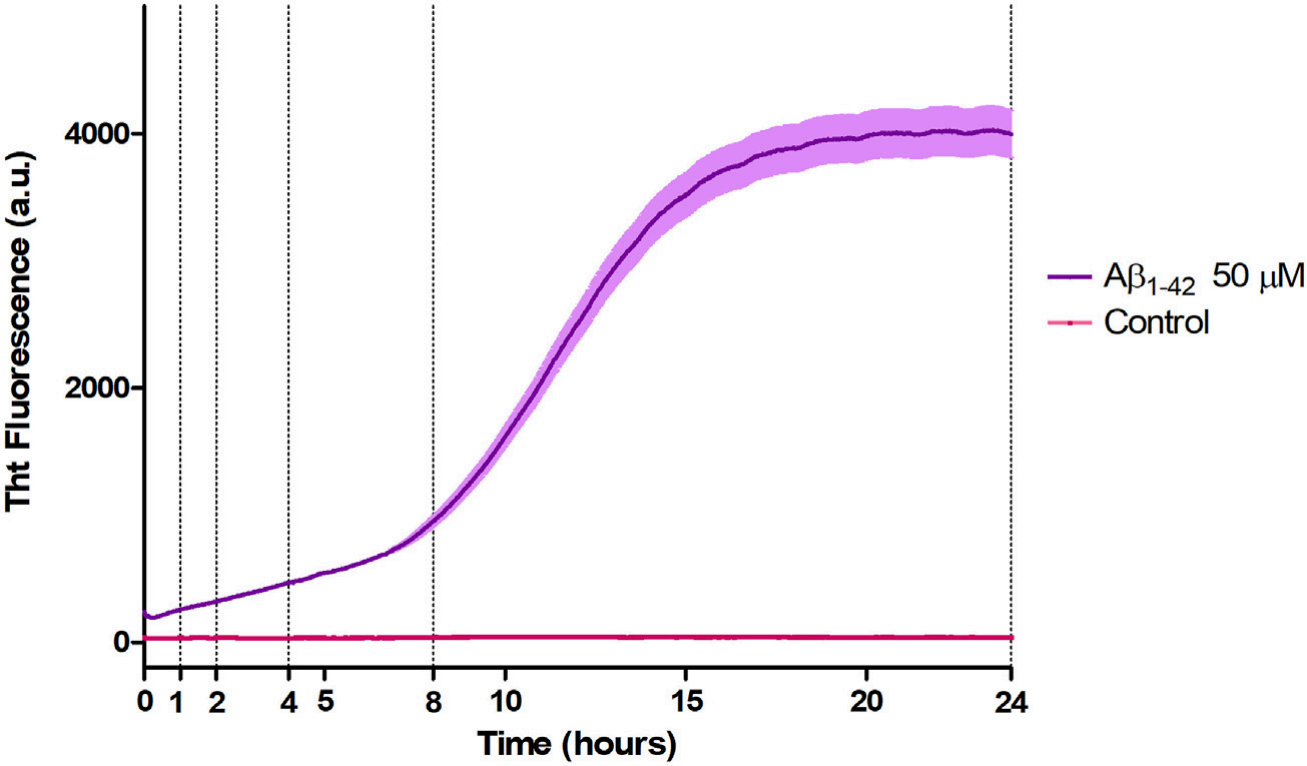
Aggregation kinetic of Aβ_1-42_ (purple) *versus* control (fuchsia) monitored by ThT fluorescence assay (λex = 440 nm, λem = 485 nm).

Aβ_1-42_ samples showed a higher fluorescence signal since the initial incubation times with respect to the control (Figure 4), compatible with the presence of small assemblies revealed by AFM imaging (Figure 1) and in agreement with previous studies (Blackley et al., 1999; 2000; Banchelli et al., 2020). Furthermore, the ThT fluorescence signal slightly increased up to 8 h of incubation indicating a slow and progressive reorganization of small and flexible aggregates. After 8 h of incubation, we observed the beginning of the exponential (or elongation) phase, indicating an increase in the number of β-sheet structures and size of rigid filaments, consistent with AFM imaging. From 15 h, the ThT assay has reached a plateau indicating the presence of mature fibrils.

### 3.3 SERS

**FIGURE 5:**
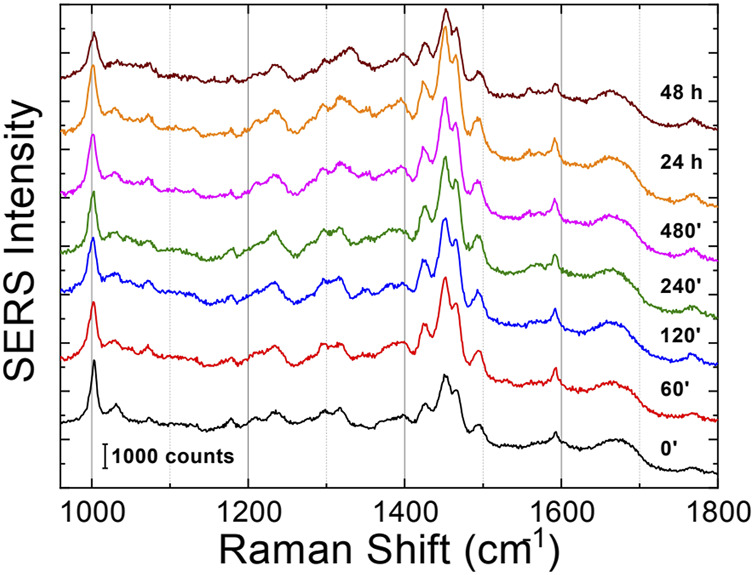
SERS averaged spectra (λex = 633 nm) of Aβ_1-42_ that was deposited on AgNWs substrate at different time points: 0 min, 60 min, 120 min, 240 min, 480 min, 24 h and 48 h. Each spectrum was calculated as average from 50 acquisitions. The spectra were staked for clarity.

**FIGURE 6:**
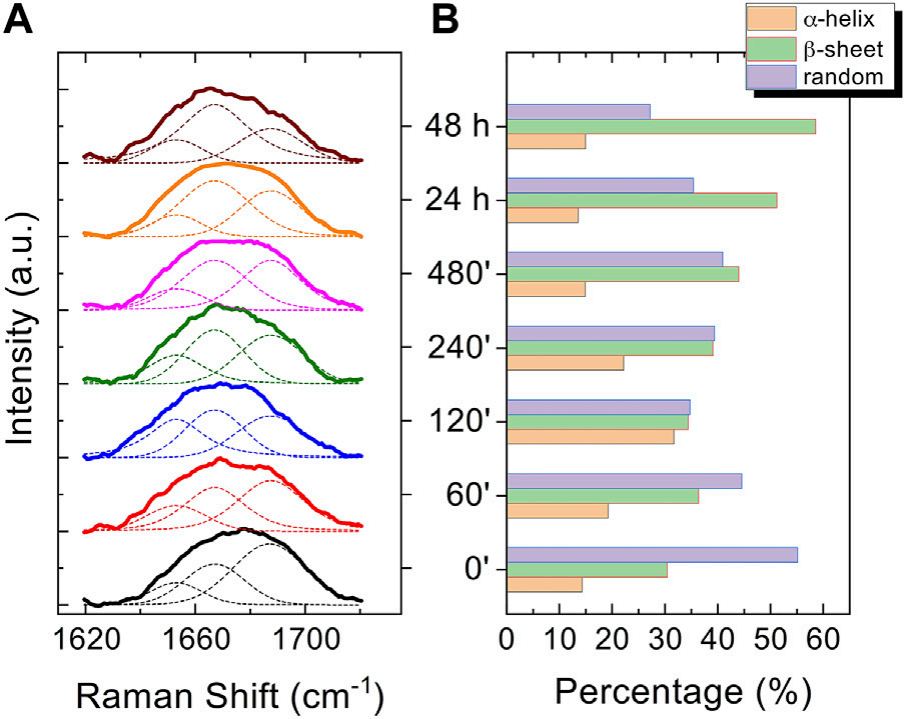
Normalized curve fitting of the amide I vibration mode of Aβ_1-42_ aggregates at different time points: 0 min, 60 min, 120 min, 240 min, 480 min, 24 h and 48 h. Three shoulder bands approximately centred at 1650, 1670 and 1680 cm^-1^, associated with α-helix, β-sheet and random coil structures, respectively, are obtained from the fitting procedure (dashed lines) **(A)**. Histogram displaying the percentage contribution of each secondary structure (α-helix, β-sheet, and random coil) to the amide I vibration band **(B)**.

The Raman enhanced spectra in the region of 950-1800 cm^-1^ of Aβ_1-42_ in PBS acquired on the samples at different incubation times are shown in Figure 5. Vibrational bands associated with aromatic amino acid residues (Phe, Tyr) at 1003, 1032, 1592 cm^-1^, CN stretching modes of amino-terminated amino acids spanning 1047-1130 cm^-1^, and CH_2_/CH_3_ deformations of side chains of hydrophobic amino acids, from 1420 to 1468 cm^-1^, are distinguishable against background signals (996, 1178-1330 cm^-1^). Additionally, the spectra reveal bands relative to amide groups in the 1349-1353 cm^-1^, 1555-1560 cm^-1^ and 1635-1710 cm^-1^ regions, assigned to components of amide III, amide II and amide I, respectively (Banchelli et al., 2020; Lipiec et al., 2021). Even though the intensity of the amide II and amide III bands exhibits a slight increase at longer incubation times, they were not considered in the spectral analysis due to overlapping with background signals. In contrast, the broad amide I band around 1635-1710 cm^-1^, with its asymmetric shape and width, was analyzed as an indicator of the distribution of secondary structures. Since the Raman spectra are affected by the Φ and Ψ angles of each amino-acid residue, the H-bonding pattern, and the peptide-peptide dipole coupling, information on the secondary structure is reflected in the Raman amide I band region, which has a major contribution from C=O stretching (Miura and Thomas, 1995). The band fitting of the amide I revealed three shoulder bands approximately centered at 1650, 1670 and 1680 cm^−1^ (Figure 6A), associated with α-helix, β-sheet and random coil structures, respectively (Shao et al., 1999; Maiti et al., 2004). Thus, the percentage contribution of each secondary structure (α-helix, β-sheet, and random coil) to the amide I band can be used to obtain a semi-quantitative assessment of the secondary structure composition. The fitting of the amide I vibration mode of Aβ_1-42_ showed that in the initial stages of incubation the major contribution comes from the random coil structural conformation (Figure 6A). The contribution of β-sheet and random coil is almost equivalent at 120 and 240 min of incubation, but at 480 min we observe a clear transition, in which the β-sheet conformation becomes the main contributor. The α-helix contribution rises at first, reaching a high at 120 min, possibly at the “expense” of the random coil conformation, and then decreases again.

## 4 Discussion

In the past few years extensive effort has been made to identify the structural determinants of the amyloid-β aggregates that are responsible for their ability to induce neurodegeneration (Hardy and Higgins, 1992; Haass and Selkoe, 2007; Benilova et al., 2012; Chiti and Dobson, 2017; Bigi et al., 2022; Limbocker et al., 2023). In this study, we employed AFM and SERS to examine the Aβ_1-42_ fibrillogenesis, studying the aggregation intermediates morphologically and monitoring spectroscopically the gradual changes in their secondary structures, throughout the course of the aggregation process. We identified oligomers and small protofibrils since the first incubation time-points. This finding is in accordance with the species observed by Blackley et al. by *in-situ* AFM during the first 135 min of incubation (Blackley et al., 1999; Blackley et al., 2000). Furthermore, we observed that such protofibrils, interact with each other forming large macromolecular networks (Figures 1C, 3A). Interestingly, at 24 h of incubation the mature amyloid-β fibrils were frequently found lying above such structures (as shown in Figure 3A panel on the bottom left) and this could indicate that these networks of protofibrils play a role in the development of mature fibrils. The physiological and physicochemical aspects driving their formation, such as the types of interactions that take place between the protofibrils, should be further investigated, in order to elucidate their role in the aggregation pathway. Our study supports the idea of the existence of different aggregation pathways, as observed in previous studies (Jiang et al., 2012; Lipiec et al., 2018), since we detected both fibrillar structures and amorphous aggregates during all the incubation time-points. Ultimately, we were able to monitor the gradual change in the secondary structure of the aggregation species by fitting the amide I band of the SERS spectra. We tracked the change from a conformation in which the random coil had a prominent contribution, in the early stages of incubation, to β-sheet-rich structures, which become the main contributors at long incubation times. SERS results show that the morphological differences between the first rigid filaments, formed between 240’ and 480’ (Figure 2) and the earlier flexible protofibrils, formed at 60’ and 120’ (Figure 1), reflect a critical conformational change (Figure 6) and lead to the formation of mature fibrils at 24 h (Figure 3). Remarkably, the exponential (or elongation) phase begins after 8 h of incubation at 25°C (Figure 4), concurrently with the structural transition observed at 480’ by SERS, when the β-sheet conformation becomes the main contributor (Figure 6). This trend is in accordance with previous findings reporting that the amyloid-β aggregation pathway is characterized by the transition of Aβ peptides from their soluble forms into disease-associated β-sheet-rich conformers (Janek et al., 2001; Chiti and Dobson, 2017). The contribution of the α-helix initially increases, peaking at 120’, concomitantly to the decrease of the random coil conformation, and then gradually goes down. This pattern could be explained by the structural rearrangements taking place during the different stages of the amyloid-β aggregation. The Aβ_1-42_ monomer contains two α-helices: S8-V24 and 28 K28-V38 (Janek et al., 2001; Crescenzi et al., 2002; Santoro et al., 2021). The α-helical structures are likely more exposed during the first 120’ of incubation, when the sample contains small-size early aggregation species, and exhibit a stronger SERS signal, with respect to the later stages of incubation, when the transition into larger β-sheet-rich species occurs. Such results align with the Aβ_1-42_ aggregation kinetics described in previous circular dichroism investigations (Bartolini et al., 2007; Jiang et al., 2012; De Simone et al., 2019); slight variations may arise due to different protein concentrations and aggregation protocols, but overall trends are in agreement.

## 5 Conclusion

In this study, we provide the significance of investigating the early phases of fibrillogenesis, to better understand the molecular pathophysiology of AD and identify potential pharmaceutical targets that could prevent or slow down the aggregation process. Our findings indicate that there is a peculiar step in the fibrillogenesis timeline when protofibrils serve as a template for the formation of larger size fibrils, which is characterized by a transition in the conformation of the secondary structure. In fact, when AFM topography starts to reveal the presence of such larger size fibrils, the organized β-sheet-rich structures spectroscopically prevail over the random coil conformation. The critical role of protofibrils *in vitro* strongly suggests that they might be crucial in the formation of amyloid fibrils in the brains of AD patients as well. Thus, the development of new therapeutic molecules able to inhibit the protofibril to fibril transition *in vitro* might lead the way for the development of therapies against AD. Finally, we highlighted how a direct, label-free and fast optical technique such as SERS, can be exploited for chemo-structural investigation of the Aβ_1-42_ aggregation process, as well as for other misfolded proteins, using minimal volumes of sample.

## Data Availability

The original contributions presented in the study are included in the article/supplementary material, further inquiries can be directed to the corresponding authors.
